# Toxicity Profile of a Nutraceutical Formulation Derived from Green Mussel *Perna viridis*

**DOI:** 10.1155/2014/471565

**Published:** 2014-06-09

**Authors:** Kajal Chakraborty, Deepu Joseph, Selsa J. Chakkalakal

**Affiliations:** Marine Biotechnology Division, Central Marine Fisheries Research Institute, Ernakulam North P.O., PB No. 1603, Cochin, Kerala 682018, India

## Abstract

The short-term (acute) and long-term (subchronic) toxicity profile, mean lethal dose 50 (LD_50_), and no-observed-adverse-effect level (NOAEL) of a nutraceutical formulation developed from green mussel *Perna viridis*, which showed *in vitro* and *in vivo* anti-inflammatory properties, were evaluated in the present study. The formulation was administered to the male and female Wistar rats at graded doses (0.5, 1.0, and 2.5 g/kg body weight) for two weeks of acute toxicity study and 0.5, 1.0, and 2.0 g/kg body weight for 90 days in subchronic toxicity study. The LD_50_, variations in clinical signs, changes in body weight, body weight, food/water consumption, organ weight (liver, kidney, spleen, and brain), hematology, serum chemistry, and histopathological changes were evaluated. The LD_50_ of the formulation was 5,000 mg/kg BW. No test article related mortalities as well as change in body weight, and food and water consumption were observed. No toxicity related significant changes were noted in renal/hepatic function, hematological indices, and serum biochemical parameters between the control and treated groups. Histopathological alterations were not observed in the vital organs of rats. The subchronic NOAEL for the formulation in rats is greater than 2000 mg/kg. This study demonstrated that the green mussel formulation is safe to consume without any adverse effects in the body.

## 1. Introduction

Bivalves are considered vital next to fish and prawns from the nutritive point of view. Bivalve molluscs were reported to contain bioactive lipids, which include fatty acids: sphingolipids, phytosterols, diacylglycerols, and so forth. And many of these can influence human health and disease linked to alleviating the symptoms of inflammatory conditions [1]. The green mussel* Perna viridis* (family: Mytilidae) is a bivalve mollusc native of the Indian coast and throughout the Indo-Pacific and Asia-Pacific [2]. It forms a significant fishery and contributes nearly 50% to the total bivalve production of the area [3].

Among the marine invertebrates, the molluscs are a potential source of bioactive substances with antitumor, antileukaemic, anti-inflammatory, antibacterial, and antiviral activities [4, 5]. Traditionally, indigenous people, notably in Western Mexico and throughout the South Pacific, use shellfish supplements as a remedy for arthritis [6]. The commercially available products, namely, freeze-dried extract (Seatone) and CO_2_ extracted oil (Lyprinol), obtained from* Perna canaliculus* were reported to inhibit inflammation in the treatment of rheumatoid arthritis and osteoarthritis [7]. Okinawan mollusc* Pinna muricata* contains aconstituent, pinnatoxin A, which is reported to have Ca^2+^ channel activating and anti-inflammatory properties [8]. New Zealand green-lipped mussel* P. canaliculus* and the Tasmanian blue mussel* Mytilus galloprovincialis* have been reported to possess anti-inflammatory components [9].* P. canaliculus* is restricted to the temperate waters around New Zealand, whereas* Perna viridis* occurs widely in tropical waters throughout the Indo-Pacific region [10].

There are several drugs like NSAIDs (aceclofenac, diclofenac, etc.), steroids (glucocorticoid), DMARDs (methotrexate and cyclosporin A), and coxibs (celecoxib and rofecoxib) for managing moderate to severe cases of arthritic pain, stiffness, and inflammation [11]. However, the side effects of these drugs are often deleterious, which include gastrointestinal ulcers, cardiovascular diseases, and reported toxic effects on the vital organs in the body [12].

The* in vitro* and* in vivo* anti-inflammatory studies of the green mussel derived nutraceutical formulation showed that green mussel* Perna viridis* contains anti-inflammatory ingredients which can be useful against inflammatory pain. With the interesting pharmacological properties of the said formulation, it has become imperative that the anti-inflammatory preparation is evaluated for its toxicity profile. As a part of the safety evaluation of this nutraceutical formulation, the present study was carried out to determine the changes in body weight, food and water consumption, hematological parameters, serum biochemistry, and histopathological changes as indices of toxicosis with the aim of providing guidance for selecting a safe dose of its use. The acute oral toxicity study in 14 days was carried out at a very high dose, whereas the repeated dose 90-day oral toxicity study was performed to establish the no-observed-adverse-effect level (NOAEL) of the extract as parts of a safety assessment according to the internationally accepted guidelines.

## 2. Materials and Methods

### 2.1. Animals

The toxicity studies and anti-inflammatory study were conducted in adult Wistar rats (both males and females; 180–300 g; 7-8 weeks old) purchased from Sri Venkateshwara Enterprises, Bangalore. The animals were housed in well ventilated polypropylene cages under controlled temperature (22–25°C), pressure, relative humidity (60–80%), and light/dark cycle of 12 h under normal laboratory conditions (24–26°C and 60–75% RH), under a 12 h light/dark cycle by fasting with distilled water. They were provided with animal feed (Sai Durga Feeds and Foods, Bangalore, India) and water* ad libitum*. All animal experiments were conducted after getting prior permission from the Institutional Animal Ethics Committee and as per the instructions prescribed by the Committee for the Purpose of Control of Supervision of Experiments on Animal (CPCSEA), Ministry of Environment and Forest, Government of India.

### 2.2. Test Article and Evaluation of Anti-Inflammatory Activities

The test article is a nutraceutical formulation prepared from the green mussels (*Perna viridis*), and the detailed collection of the raw material, processing, method(s) used to assure stability under storage conditions, and chemical analysis demonstrating the composition of the material have been described elsewhere [5]. Briefly, the meat (3 kg) from the samples of green mussel (*P. viridis*) (10 kg) collected from their natural habitat at Elathur (Lat: 11054′11.6′′N; Long: 75012′21.8′′E) in the southwest coast of India (Kerala state) has been sucked, homogenized, and lyophilized to get the freeze-dried green mussel extract (214 g; yield 7.13%). The content, thus, prepared has been added with lysolecithin, substituted polysaccharides, and phenolic derivatives isolated from* Perna viridis*. In order to enhance the stability and activity of the freeze-dried green mussel extract several natural sources of antioxidant additives, oleoresins of* R. officinalis* (0.4%) and* C. longa* (0.8%), trace amounts of other additives,* namely*, aqueous freeze-dried extracts of marine macroalgae (*Turbinaria conoides* and* Sargassum myriocystum*, 0.025% w/w),* Zingiber officinale, Tamarindus indica, Emblica officinalis, Citrus limon*, and* Ananas comosus* (0.05% w/w), were selected and added to the freeze-dried extract to make the green mussel nutraceutical formulation.

The* in vitro* anti-inflammatory activities of the green mussel formulation have been carried out in this study using cyclooxygenase (COX_I_ and COX_II_) inhibition assays by 2,7-dichlorofluorescein method [13] and the 5-lipoxygenase (LOX_V_) inhibition assay [14]. For COX_I_ and COX_II_ inhibition assays, leuco-2,7-dichlorofluorescein diacetate (5 mg) was hydrolysed at RT in 1 M NaOH (50 *μ*L) for 10 min; then 1 M HCl (30 *μ*L) was added to neutralise the excess of NaOH before the resulting 1- dichlorofluorescein (DCF) was diluted in 0.1 M Tris-buffer (pH 8). COX enzyme (COX_I_ and COX_II_) was diluted in 0.1 M Tris-buffer (pH 8), so that a known aliquot gave an absorbance change of 0.05/min in the test reaction. Test samples (or the equivalent volume of MeOH, 20 *μ*L) were preincubated with the enzymes at RT for 5 min in the presence of hematin. Premixed phenol, 1-DCF, and arachidonic acid were added to the enzyme mixture to begin the reaction and to give a final reaction mixture of arachidonic acid (50 *μ*M), phenol (500 *μ*M), 1-DCF (20 *μ*M), and hematin (1 *μ*M) in 1 mL final volume of 0.1 M Tris-buffer (pH 8). The reaction was recorded spectrophotometrically over 1 min at 502 nm. A blank reaction mixture (without enzyme) was analysed in the spectrophotometer reference cell against each test reaction to account for any nonenzymatic activity attributed to the test sample. For 5-lipoxygenase (LOX_V_) inhibition assay, an aliquot of the stock solution (50 *μ*L, in DMSO and tween 20 mixture; 29 : 1, w/w) of each sample was placed in a 3 mL cuvette, followed by prewarmed 0.1 M potassium phosphate buffer (2.95 mL, pH 6.3) and linoleic acid solution (48 *μ*L). Thereafter, ice-cold buffer (potassium phosphate) (12 *μ*L) was mixed with LOX_V_ enzyme (100 U). The mixture was then transferred to the cuvette, shaken, and placed into the spectrophotometer, before the absorbance was recorded at 234 nm. It is important to note that, prior to testing the sample, two samples were prepared as mentioned above but only with DMSO and Tween 20 mixtures, to serve as controls (no enzyme inhibition).

The* in vivo* anti-inflammatory activity of the green mussel formulation was carried out using the carrageenan-induced rat paw edema as described elsewhere [15]. Thirty minutes after oral administration of the samples (250 mg/kg animal) and reference drug (aspirin, 200 mg/kg animal), in normal saline, an injection of 0.1 mL of carrageenan (1% in normal saline) was made into the subcutaneous portion of right hand paw of each animal. The paw thickness was measured using an electronic micrometer (aerospace; 0–25 mm range, least count: 0.001 mm) immediately before carrageenan injection and 2, 3, 4, 5, and 6 h after carrageenan injection. Percentage (% difference in paw edema compared to control group) was obtained using the following formula: (*T*_*t*_ − *T*_0_) × 100/*T*_0_, where *T*_*t*_ is the average thickness obtained for each group before any treatment (0th h) and *T*_0_ is the average paw thickness for each group after treatment in different time intervals (2, 3, 4, 5, and 6 h).

### 2.3. Lethal Dose 50 (LD_50_) of Green Mussel Formulation

Fifteen animals were divided randomly into three groups containing 5 animals each. After being fasted for 16 h, the animals were administered different doses of green mussel formulation suspended in distilled water (5000, 2500, and 1500 mg/kg BW) and administered as a single dose through oral gavage. The animals were monitored for 14 days for mortality, clinical and behavioral symptoms, and any adverse reaction.

### 2.4. Acute Oral Toxicity Study of Green Mussel Formulation

Forty animals (20 males and 20 females) were divided into 4 groups, each consisting of 5 male and 5 female rats, and three doses (2.5, 1.0, and 0.5 g/kg) of the green mussel formulation were administered orally (once daily) for 14 days. The control received 1 mL of water as vehicle every day. The animals were monitored for mortality, clinical symptoms, and any adverse reaction of the test material. The body weight and food consumption were determined in different time intervals (0, 14, 42, 70 and 91 days). After 14 days, the animals were sacrificed under mild ether anesthesia, and the blood was collected by direct heart puncture method. Necropsy was performed and observations were recorded. Selected organs such as the liver, kidney, brain, and spleen were dissected out, weights were recorded, and histopathological analyses were performed.

### 2.5. Subchronic Oral Toxicity Study of Green Mussel Formulation

Forty animals (20 males and 20 females) were divided into 4 groups, each consisting of 5 male and 5 female rats, and three doses (2.0, 1.0, and 0.5 g/kg) of the green mussel formulation (1 g suspended in 6 mL double distilled water) were administered orally (once daily) for 90 days [16]. The control received 1 mL of water as vehicle every day. The test animals were monitored, during this period for any type of clinical symptoms, mortality, and adverse reaction. The body weight and food consumption were determined every seven days. On the 91st day, the animals were sacrificed under mild ether anesthesia. Blood was collected by direct heart puncture method. Necropsy was performed and observations were recorded. Selected organs such as the brain, kidney, liver, and spleen were dissected out, weights were recorded, and histopathological analyses were performed.

### 2.6. Hematology and Clinical Chemistry Parameters

Blood collected in EDTA tubes was analyzed for hematological parameters [17]. Red blood cell (RBC), total white blood cell count (WBC), platelet count, and hemoglobin (HGB) were determined using a haematology analyzer (Model-Diatron, 9 Wein, Austria). Total white blood cells were measured after diluting the blood in Turk's fluid and counting them using a hemocytometer [18]. For differential counts (lymphocytes, eosinophils, and neutrophils) blood was spread on a clean slide and treated with Leishman's stain before being counted manually with a microscope (100x) [19].

A part of the blood was collected in nonheparinized tubes and serum was separated after centrifugation at 5000 rpm for 10 min which was used for the following investigations. Serum glutamic oxaloacetic transaminase (SGOT) and serum glutamic pyruvic transaminase (SGPT) were assayed according to the method described by Bergmeyer et al. [20]. Alkaline phosphatase (ALP) was estimated by p-nitrophenyl phosphate (PNPP) hydrolysis [21]. Total bilirubin was determined by Jendrassik-Diazotized sulphanilic acid method [22]. The total protein concentration was determined by biuret method [18]. Albumin was determined based on its reaction with bromocresol green. Markers of kidney function such as creatinine and blood urea nitrogen were estimated by Jaffe-Kinetic and urease method, respectively [23]. Serum sodium, potassium, and bicarbonate were estimated using Flame photometer 129 ion selective electrolyte analyzer. Chloride was estimated by mercuric thiocyanate method using a kit from Raichem Lifesciences Pvt Ltd, India. Total cholesterol was estimated by CHOD–PAP (cholesterol oxidase–phenol + aminophenazone) enzymatic method [24]. Triglyceride was estimated by GPO–PAP (glycerol-3-phosphate oxidase–phenol + aminophenazone) method [25], and high-density lipoprotein (HDL) was determined after precipitation with phosphotungstic acid. Very low-density lipoprotein (VLDL) was estimated by the Friedewald equation (VLDL = triglyceride/5) and low-density lipoprotein (LDL) by calculation: LDL = total cholesterol − (HDL + VLDL) [16].

### 2.7. Histopathological Analysis

A portion of the selected organs (brain, kidney, liver, and spleen) of control and treated group (high dose groups) were fixed in 10% neutral buffered formalin. Embedded organs tissue samples were cut into slices of 2–4 *μ*m and stained with hematoxylin-eosin, and the sections were observed under light microscope (40x).

### 2.8. Statistical Analysis

Statistical evaluation was carried out with the Statistical Program for Social Sciences 13.0 (SPSS Inc, Chicago, USA, ver. 13.0). Analyses were carried out in triplicate, and the means of all parameters were examined for significance by analysis of variance (ANOVA). The values were compared with that of untreated control animals.

## 3. Results

### 3.1. Anti-Inflammatory Activities of Green Mussel Formulation

**Figure 1:**
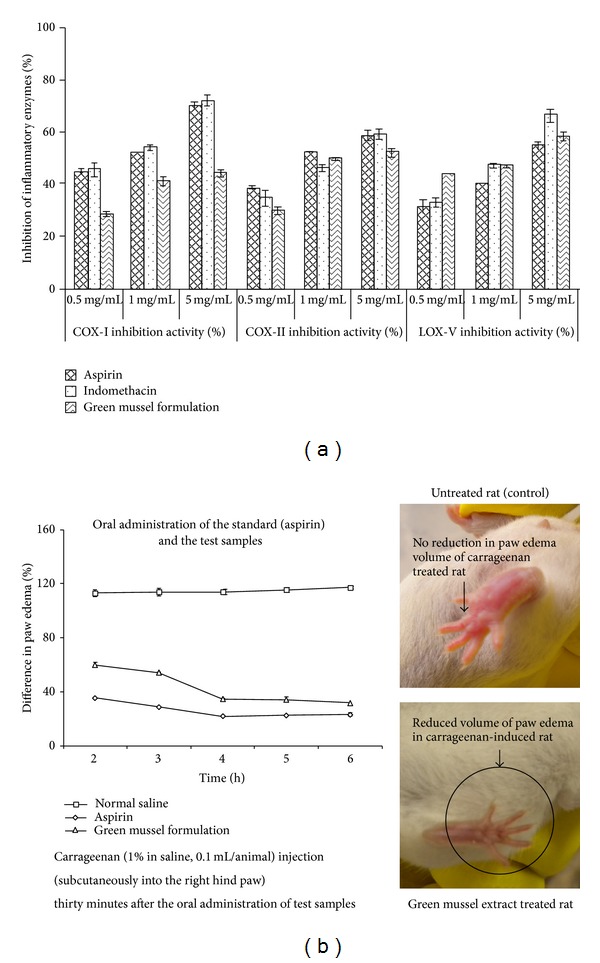
(a)* In vitro* anti-inflammatory activities (COX_I, II_ and LOX_V_ inhibition activities) of the green mussel formulation compared with standard anti-inflammatory drugs, aspirin and indomethacin, at different concentrations (0.5, 1, and 5 mg/mL). (b)* In vivo* anti-inflammatory activity (% difference in paw edema compared to control group) of the green mussel formulation compared to standard anti-inflammatory drug, aspirin.

The green mussel formulation (1 mg/mL) showed inhibiting properties against proinflammatory COX_II_ (50%) and LOX_V_ enzymes (47%), and the activities were found to be comparable with standard NSAIDs (Figure 1(a)). In this study, green mussel formulation showed lower inhibition of COX_I_ (41%, 1 mg/mL) than synthetic NSAIDs (>50%). Notably, the animals challenged with the green mussel formulation significantly mitigated (*P* < 0.05) the carrageenan-induced paw edema in a time-dependent manner till the end of the 6th h as compared to negative control animals throughout the period of study (Figure 1(b)).

### 3.2. LD_50_ of Green Mussel Formulation

The single dose administration of the green mussel formulation up to a concentration of 5000 mg/kg BW did not produce any mortality after 14 days of observation, which indicates that the mean lethal dose (LD_50_) of the formulation is greater than 5000 mg/kg BW. The oral toxicity of this formulation can be classified in category 5 (the lethal acute toxicity is greater than 5000 mg/kg) according to the Globally Harmonized Classification System of OECD [26].

### 3.3. Acute Toxicity Study of Green Mussel Formulation

No treatment-related signs of mortality were observed in the animals over short-term administration (maximum dose of 2500 mg/kg BW). In addition, the administration of the green mussel formulation at different doses did not produce any treatment-related changes in the body weight of the animals or any differences in the food consumption of male and female rats when compared to controls. No significant changes were noticed during necropsy and there was no change in the organ weight.

No treatment-related biologically significant effects of the green mussel formulation treatment at dose levels of 0.5–2.5 g/kg in hematology parameters such as hemoglobin, RBC count, platelet count, and total and differential leukocytes counts were apparent in both genders of rats when compared to untreated animals.

The green mussel formulation up to a concentration of 2.5 g/kg did not produce any change in the hepatic function parameters in serum such as SGOT, SGPT, ALP, total protein, bilirubin, albumin, and globulinas well as in albumin/globulin (A/G) ratio.

The renal function tests such as blood urea and serum creatinine did not show any variation when compared to controls. There was also no change in serum electrolytes sodium, potassium, chloride, and bicarbonate indicating that the green mussel formulation did not produce any change in renal function.

Acute toxicity study of the green mussel formulation did not show any change in cholesterol, triglycerides, HDL, LDL, VLDL, and cholesterol levels. Histopathological analysis of the brain, spleen, kidney, and liver did not show any pathological lesions in the organs of animals treated with the green mussel formulation.

The above observations concluded that the green mussel formulation did not produce any toxicity to Wistar rats when administered for two weeks.

### 3.4. Subchronic Toxicity Study of Green Mussel Formulation

#### 3.4.1. General Conditions and Behavior

No treatment-related signs of mortality were observed in the animals over the administration periods (maximum dose of 2000 mg/kg BW). In addition, the administration of the green mussel formulation at different doses did not produce any treatment-related changes in clinical signs such as mental state, external appearance, and daily activities among the test groups when compared with the control. Any abnormal behavior or cases of diarrhea and soft feces were not observed during the period of study. No ophthalmological abnormalities were observed in any of the treatment groups prior to study initiation and near experimental completion. In general, the experimental animals from all treatment groups appeared healthy at the conclusion of the study period and did not induce any clinical signs of toxicity in subchronic regimens.

#### 3.4.2. Body Weight

The administration of the green mussel formulation during 90 days of long-term subchronic toxicity studies did not produce any abnormal change in the body weight of male and female rats when compared to the control (Table 1). As expected, rats gained weight with time. In male rats, the gain in mean body weights for the treated groups at 0.5–2.0 g/kg BW was comparable with those in the control group throughout the study. Similarly, the mean body weights of the treated female rats were comparable with those in the control group throughout the study. There were no changes in body weight in the animals attributable to the administration of the green mussel formulation when compared to the control group. Any changes observed were sporadic, considered incidental, and unassociated with test article administration.

#### 3.4.3. Food and Water Consumption

The average food intake of untreated control rats decreased from about 72 g to 68 g (for male rats) and from 60 g to 49 g (for female rats) after 90 days of study. The same trend was observed for medium and low dose group (1.0 and 0.5 g/kg, resp.) of males and all the dose groups of females. Administration of the green mussel formulation did not produce any significant difference in the food consumption of both genders of rats when compared to normal animals of the high dose group (*P* > 0.05) throughout the experimental period. The summarized food intake of the rats recorded after oral administration of the green mussel formulation to rats is shown in Table 1. Similarly, the water consumption did not alter in male and female rats attributable to administration of the green mussel formulation when compared to normal animals during chronic and subchronic toxicity studies. Changes in the average water consumption during the treatment period are presented in Table 1. Sporadic statistically significant changes in water consumption were considered spurious, unassociated with the test article administration.

#### 3.4.4. Relative Organ Weight

**Figure 2:**
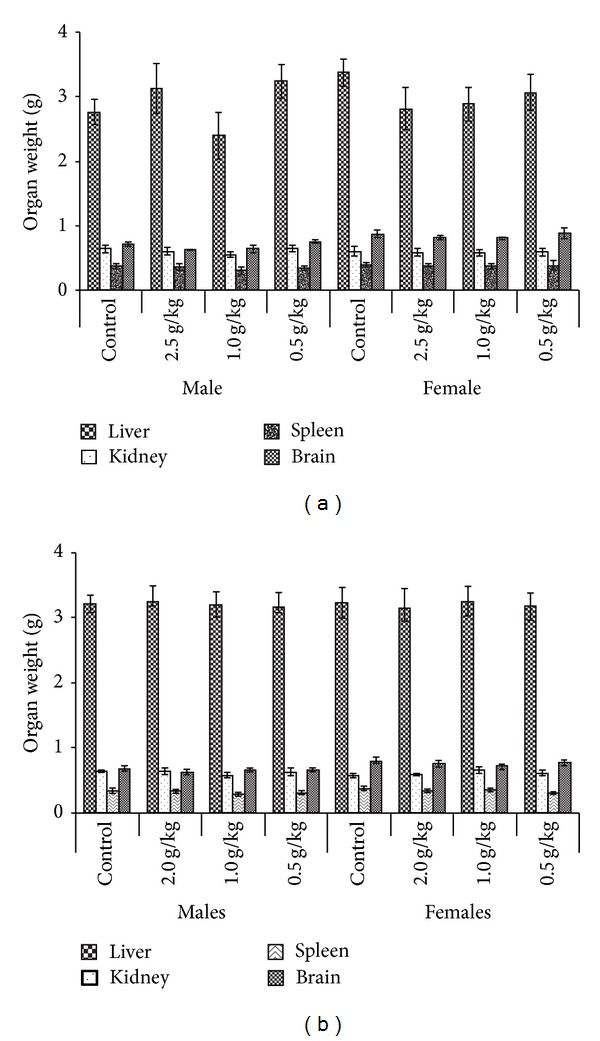
Mean organ weights (in grams) of male and female rats administered green mussel formulation after (a) acute and (b) subchronic toxicity studies.

Figure 2 presents the relative weights of the vital organs (in g) of rats (both genders). The weights of liver, kidney, spleen, and brain recorded at the end of the subchronic study (day 91) did not show significant differences (*P* > 0.05) in any of the treatment groups compared with the control groups (Figure 2). Furthermore, gross examination of the vital organs of all rats revealed no detectable abnormalities.

#### 3.4.5. Hematological Parameters

The effect of the green mussel formulation on hematological parameters such as hemoglobin (HGB), RBC and WBC count, platelet count, and differential counts after subchronic toxicity (90 days) studies is presented in Table 2. No treatment-related biologically significant effects of the green mussel formulation treatment at dose levels of 0.5–2.0 g/kg in the hemoglobin and RBC and platelet content were apparent in both genders of rats when compared to untreated animals (Table 2) (*P* > 0.05) and remained within physiological range throughout the treatment period (90 days). However, both male and female rats administered green mussel formulation (at 1.0 g/kg and 0.5 g/kg, resp.) showed significantly low levels of differential counts (lymphocytes, eosinophils, and neutrophils) compared to control animals (*P* < 0.05). Similarly, female rats administered 2.0 and 1.0 g/kg green mussel formulation recorded significantly low lymphocyte and neutrophil count, respectively (*P* < 0.05). No test article-related changes in blood cell morphology were observed during the period of study.

#### 3.4.6. Serum Biochemical Parameters

Table 3 summarizes the serum biochemical parameters used as the biomarkers of the liver and renal functioning, during the course of subchronic toxicity studies. The serum analysis showed significantly low SGOT content for the low dose male rats (0.5 g/kg) and high/low dose female rats (2.5 and 0.5 g/kg) after 90 days of subchronic study. Similarly, another marker enzyme of the liver, ALP, also showed significantly low values for high and low dose group male rats compared to the control animals after 90 days of subchronic study. The activity of another marker enzyme SGPT was not significantly different (*P* > 0.05) in all dose groups of treated rats as compared to untreated control, and albumin/globulin (A/G) ratio was not altered in the treated animals of both genders (Table 3).

Subchronic oral administration of the green mussel formulation (for 90 days) did not cause any significant changes in hepatic function parameters such as total protein, albumin, total bilirubin, and globulin in both sexes of rats during long-term subchronic toxicity studies. The renal function parameters such as serum creatinine and blood urea did not show any significant variation (*P* > 0.05) in treated animals compared to controls (Table 3). There were no statistically significant differences in the levels of serum electrolytes such as chloride, potassium, sodium, and bicarbonate after the treatment of green mussel formulation (*P* > 0.05), indicating no expressive changes in the general metabolism after consumption of the green mussel formulation by rats (Table 4). The green mussel formulation did not produce any significant changes in the total cholesterol, HDL, LDL, and VLDL, indicating no expressive changes in the general lipid metabolism after consumption of the test article by rats (Table 4).

#### 3.4.7. Histopathological Analysis

**Figure 3:**
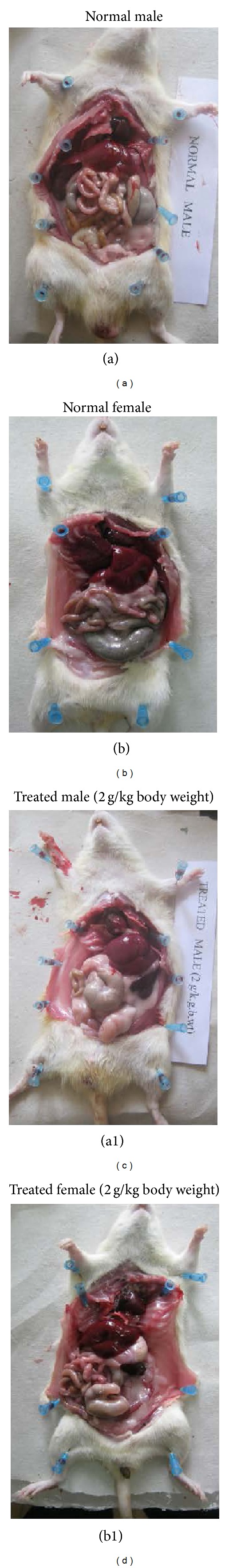
The cross section of the male and female rats after subchronic toxicity study of 90 days. (a) Normal male; (a1) green mussel formulation (2.0 g/kg) treated male; (b) normal female; (b1) green mussel formulation (2.0 g/kg) treated female.

**Figure 4:**
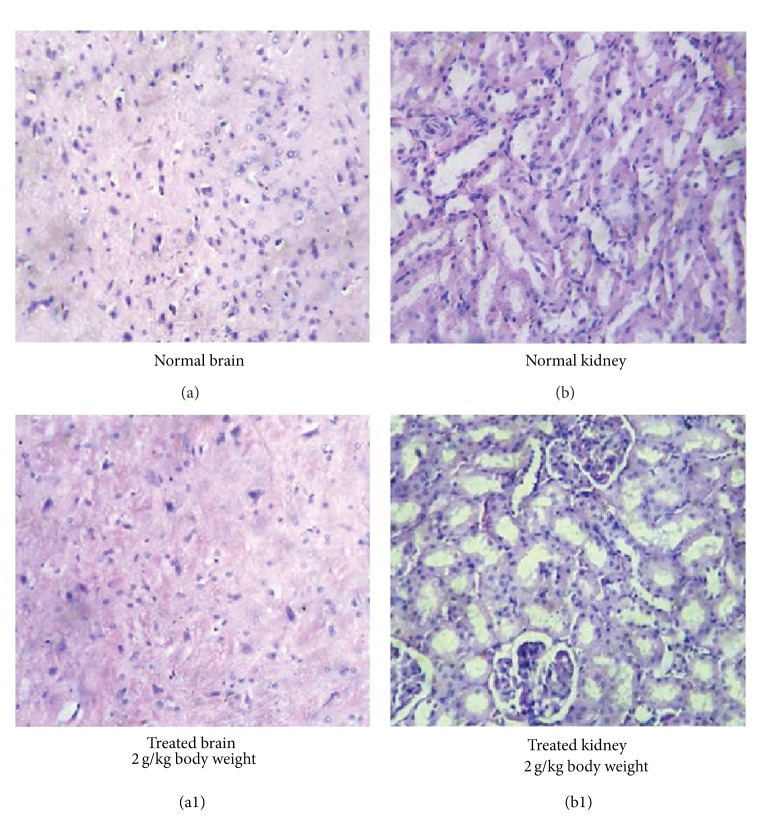
Photomicrograph of histopathological sections of the brain and kidney on day 90 of subchronic toxicity test. (a) Normal liver, (a1) brain sections from experimental rats after 90 days of treatment with 2.0 g/kg of the green mussel formulation showing apparently normal glial cells, (b) photomicrograph of kidney section from experimental control rats, and (b1) kidney sections from experimental rats after 90 days of treatment with 2.0 g/kg of the green mussel formulation showing normal glomeruli.

**Figure 5:**
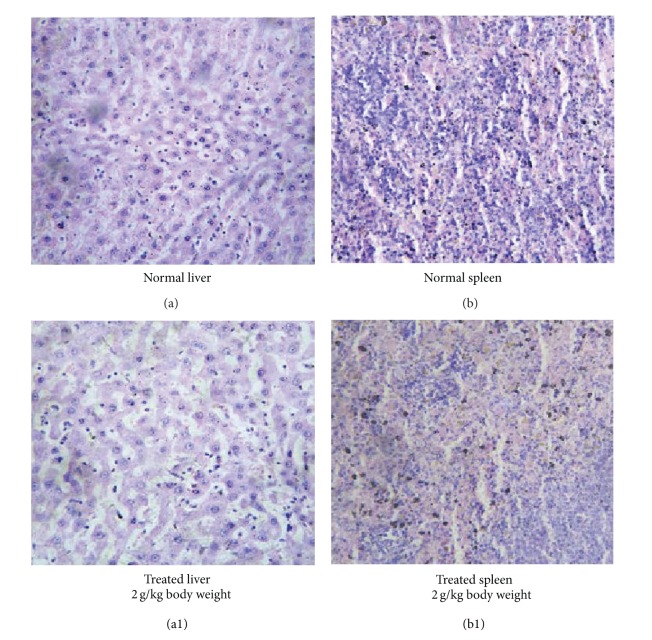
Photomicrograph of histopathological sections of the liver and spleen on day 90 of subchronic toxicity test. (a) Normal liver, (a1) photomicrograph of liver sections from experimental rats after 90 days of treatment with 2.0 g/kg of the green mussel formulation showing apparently normal morphology of hepatocytes, (b) photomicrograph of spleen section from experimental control rats, and (b1) spleen sections from experimental rats after 90 days of treatment with 2.0 g/kg of the green mussel formulation showing normal lymphoid follicles with germinal centers.

Necropsy of the treated animals after sacrifice did not show any morphological changes of the internal organs or any gross pathological abnormalities during subchronic toxicity studies. There were no macroscopic findings considered to be related to the treatment of the green mussel formulation (Figure 3). Gross examination of vital organs such as brain, kidney, spleen, and liver of rats and microscopic examination of tissue sections prepared from these organs did not observe any histopathological alteration in any treated rats during subchronic toxicity studies. The normal and treated sections of brain (Figures 4(a) and 4(a1)), kidney (Figures 4(b) and 4(b1)), liver (Figures 5(a) and 5(a1)), and spleen** (**Figures 5(b) and 5(b1)**) **showed normal appearance compared to control rats after subchronic toxicity study. The treated section of brain showed normal glial cells. Astrocytes, interstitial tissue of the brain, and the portion of cerebellum also showed normal appearance compared to control rats (Figure 4(a1)). The treated section of the kidney showed normal glomeruli with normal Bowman's capsule. Glomeruli showed normal cellularity with renal tubules and interstitial tissue demonstrated the normal appearance (Figure 4(b1)). The section of liver tissue showed normal portal triads and biliary duct. A few lymphocytic collections were seen in the portal area, which was normal. Central venous systems also appeared normal. Hepatocytes showed normal morphology and they were arranged in cords. Sinusoidal space and Kupffer cells also appeared normal (Figure 5(a1)). The section of spleen showed normal lymphoid follicles with germinal centers. Sinusoidal spaces are dilated and they were lined by normal endothelial cells. Some areas showed hemorrhaged congestion with many siderophages (Figure 5(b1)). No other macroscopic or microscopic lesions in organs examined were observed.

## 4. Discussion

The active principles in the formulation derived from green mussel* P. viridis* were competitively inhibited inflammatory cyclooxygenases (COX_I,II_) and lipoxygenase (LOX_V_) in an inflammation and oxidative stress reaction, resulting in decreased production of proinflammatory prostaglandins and leukotrienes.* In vivo* animal model studies revealed that the active principles effectively suppressed the carrageenan-induced rat paw edema, which indicate that they exhibit its anti-inflammatory action by means of inhibiting either the synthesis, release, or action of inflammatory mediators. The green mussel formulation recorded COX_I_/LOX_V_ and COX_I/II_ ratios lower than 1.0 compared to NSAIDs (>1.0), which indicate their higher selectivity against inflammatory response and lower side effect profiles.

Many of the allopathic prescriptions including NSAIDs and cyclooxygenase inhibitors used in controlling arthritic conditions have known side effects, especially with long-term usage. About 25% of the users experience some kind of side effect and 5% develop serious health consequences such as stomach bleeding, stroke, and acute renal failure. The green mussel formulation proved to be a safer and effective alternative to these synthetic NSAIDs and other products available in the market.

As it is has been observed to be slow acting, long-term use of the green mussel formulation may be required in treatments of arthritis related diseases. In this aspect, long-term toxicity studies of the green mussel formulation are of vital importance for the assessment of its safety in mammalian systems. In order to provide safety evidence for the green mussel formulation as a prospective nutraceutical medication for joint pain and arthritis, short-term and subchronic toxicity studies were conducted on the rats to evaluate the possible toxicity. The results demonstrate a lack of test substance-related general organ or systemic toxicity following oral administration of the green mussel formulation at a dose as high as 2500 mg/kg/d in the acute toxicity study and the repeated oral administration of the formulation at a dose of 2000 mg/kg/day, the highest dose tested in the 90-day oral toxicity study. According to Loomis and Hayes [27], a chemical substance with an LD_50_ within the range of 5000–15,000 mg/kg is considered as practically non-toxic. The calculated LD_50_ for the green mussel formulation is found in this range, and therefore, this nutraceutical formulation should be regarded as practically nontoxic in acute ingestion. The green mussel formulation too did not cause any toxic symptoms, behavioral changes, or mortality when acutely administered at 5000 mg/kg to rats, and therefore, this formulation can be included under category 5 (low or no toxicity) in accordance with OECD guidelines.

Under various regulatory guidelines, changes in body weight have been used as an integral part of the conventional safety evaluation of test materials, drugs, and chemicals [28, 29]. The animal behavior, feed intake, and the normal body weight changes were not altered during the short-term and subchronic toxicity studies. This indicates that the food conversion rate was not affected and growth of rats in treatment groups was comparable to that of control. Since, no significant changes were observed in the general behavior, body weight, and food and water intake of rats in the treated groups as compared to the untreated control after the administration of the green mussel formulation during the short-term and subchronic toxicity study spanning over 90-day period, it could be concluded that oral administration of this test material had no effect on the normal growth of rats in the concentration studied.

Organ weights are widely accepted in the evaluation of toxicity related studies [30]. No significant differences were recorded in the relative weights of kidney, brain, spleen, and liver indicating that the acute/subchronic oral administration of the green mussel formulation did not detrimentally affect the wet weight, organ-to-body weight ratio, and the color of the organs.

The hematopoietic system is one of the most sensitive targets of toxic compounds and is an important index to assess the toxicity of the test material on the physiological and pathological status in human and animals [31]. After 90 days, there were no treatment-related changes in hematological parameters between the untreated and the green mussel formulation treated groups indicating that the test material had no effects on the circulating blood cells, nor it interfered with their production. Some statistically insignificant differences were noted in WBC and differential counts when the control and treatment groups were compared. However, these changes did not appear to be related to the test article treatment since they were still within the limits of normal biological variation. The changes in WBC counts were probably due to the normal responses to foreign bodies or stress associated with the toxicity studies. Decreased hemoglobin and differential counts were found previously in the rats fed with polyunsaturated fatty acids, such as *n*-6 fatty acid arachidonic acid and *n*-3 fatty acid docosahexaenoic acid containing oils [32]. However, in the present study, no significant changes in these parameters were apparent. Taken together, the normal range of hematological indicators indicated the absence of hemotoxic potential of the green mussel formulation.

Biochemical determinations of blood parameters in serum serve as an indicator of toxicity of a test material [28]. The enzymes, namely, serum aspartate transaminase (AST) or serum glutamic oxaloacetic transaminase (SGOT), serum alanine transaminase (ALT) or serum glutamic pyruvic transaminase (SGPT), and alkaline phosphatase (ALP), are well-known enzymes used as good indicators of liver function and as major markers of hepatic injury [29]. In general, aminotransferases AST and ALT are normally contained within liver cells, and their activity in the blood is generally low. If the liver is damaged, these enzymes diffuse across the damaged cell membrane due to altered plasma membrane permeability [33], before being entered into the circulation, raising the enzyme levels in the blood and signaling liver disease. In the present study, there was no significant difference in ALP, ALT, and AST in the green mussel formulation administered animals when compared to control. These results suggest that the test material did not alter the hepatic function and prevent hepatocyte enzyme from going into the blood. No significant alterations in ALP of the animal subjects treated with the green mussel formulation indicated any liver injury [34]. Other liver enzyme activities too realized no significant decrease, thereby suggesting that the acute and subchronic administration of the test material did not alter the hepatocytes and consequently the metabolism of the rats.

Albumin is synthesized by the hepatocytes, and as such, it represents a major synthetic plasma protein, and its determination can act as a criterion for assessing the synthetic capacity of the liver [35]. Decrease in plasma proteins, therefore, tends to reflect chronic damage. The common pattern seen following significant hepatocellular damage is a reduction in albumin accompanied by a relative increase in globulins, which leads to A/G ratio reduction [36]. However, no change in serum proteins and albumin was observed in the acute and subchronic studies, which show that the green mussel formulation does not inhibit protein synthesis in the rats. This is supported by the microscopic examination showing the normal hepatocytes without any lesions in the liver. Bilirubin is a metabolic breakdown product of blood heme. Any course that might induce abnormally increased levels of bilirubin accumulating in human serum or plasma usually signifies the presence of a variety of diseases with liver dysfunctions, ranging from jaundice to infectious hepatitis [37]. Our results proved the report that long-term and high dose administration of the green mussel formulation did not significantly alter the bilirubin concentrations, which is an indication that it does not interfere with the metabolism of bilirubin in the liver. In liver, bilirubin also affects the protein synthesis. There was no obvious alteration of protein content in the liver at the experimental doses administered with the test material in comparison to the control group. It is of note that, under normal circumstances, bilirubin-albumin conjugate protects the cells against the potential toxicity of bilirubin. Any imbalance in the formation of the conjugate results in the decreased protein content in the liver and increased total bilirubin in serum to have a detrimental effect leading to injury of the liver. Since, there was no adverse effect on plasma levels of total bilirubin, total protein, and A/G ratio in the green mussel formulation treated animals when compared to control, it may be concluded that the test article did not alter the renal and hepatic function.

Serum urea and creatinine are known as the usual markers of renal function [35]. Any rise in their levels is only observed if there is marked damage to functional nephrons [38]. Since, there was no significant difference in plasma levels of urea and creatinine in the green mussel formulation treated animals when compared to control, it may be concluded that the test article did not alter the renal functionaries.

No significant changes were observed in cholesterol, LDL, and VLDL levels suggesting that the green mussel formulation had no effects on the lipid and carbohydrate metabolism of the rats. The microscopic evaluation of the organs of treated rats group supported the safety of the green mussel formulation. The liver is the site of cholesterol disposal and synthesis and glucose synthesis and generates free glucose into the blood from hepatic glycogen stores [39]. In the present study, lowering of triglycerides was observed, which was not significant. The present study also revealed that serum electrolytes (Na^+^, K^+^, Cl^+^, and HCO_3_^−^) did not alter significantly in the treatment groups compared to the untreated controls throughout the study period.

Most importantly, the histopathological examination of selected organs (heart, liver, brain, and kidneys) from treated and control animals showed normal architecture, suggesting that daily oral administration of the green mussel formulation for 90 days caused no detrimental changes or morphological disturbances.

The acute and subchronic toxicity studies of the green mussel formulation using Wistar rats were carried out to understand its effect on various parameters such as mortality, weight change, food consumption, hematological, liver, and renal functions, serum electrolytes, and lipid profile. The results indicated that the green mussel formulation did not produce any change in food consumption, water consumption, and body weights in rats, indicating that it has no toxicity to these animals. Also it did not produce any biochemical changes related to hepatic and renal function. This formulation did not produce any change in hematological and serum biochemical parameters. Necropsy of the treated animals showed normal appearance of various tissues. The no-observed-adverse-effect level (NOAEL) was 2000 mg/kg BW. The toxicological studies demonstrated that the green mussel formulation is safe to consume without any adverse effects in the body.

## Figures and Tables

**Table 1:** Body weight and food and water consumption during subchronic (90 days) toxicity studies after the administration of green mussel formulation.

Days	Male				Female			
	Control^a^	2.0 g/kg^b^	1.0 g/kg^b^	0.50 g/kg^b^	Control^a^	2.0 g/kg^b^	1.0 g/kg^b^	0.50 g/kg^b^
Body weight (g)								
0	230.8 ± 1.4	254.9 ± 2.5	263.6 ± 14.2	284.4 ± 0.5	185.8 ± 1.5	218.5 ± 15.6	195.8 ± 1.4	190.9 ± 5.2
14	239.6 ± 4.5	261.8 ± 1.5	270.4 ± 2.2	290.1 ± 1.4	192.4 ± 2.4	224.4 ± 12.5	202.1 ± 2.6	196.8 ± 6.2
42	252.4 ± 6.6	273.9 ± 6.5	281 ± 18.6	300 ± 1.5	204 ± 2.6	232.6 ± 11.6	212.1 ± 3.2	206.9 ± 2.6
70	263.8 ± 8.5*	283.6 ± 1.5*	290.2 ± 19.5	308.4 ± 1.6*	214.2 ± 2.6*	240.9 ± 11.4*	220.7 ± 1.2*	216.2 ± 2.4*
91	271.58 ± 14.2*	290.8 ± 1.6*	296.7 ± 2.1*	314.5 ± 1.2*	221.1 ± 0.9*	246.6 ± 0.5*	226.8 ± 0.5*	222.5 ± 2.9*

Food consumption (g)								
0	72.4 ± 5.6	74.4 ± 2.5	70.6 ± 6.5	68.7 ± 1.2	59.5 ± 0.6	55.4 ± 1.6	58 ± 2.3	62.7 ± 0.9
14	66.4 ± 6.5	73.3 ± 3.6	62.6 ± 6.3	61.3 ± 1.5	62.5 ± 1.2	49.3 ± 2.6	54.6 ± 2.6	57.7 ± 1.2
42	67.3 ± 6.9	71.6 ± 3.9	61.4 ± 4.2	60.6 ± 2.5	47.6 ± 1.1	50.6 ± 2.7	48.3 ± 1.5	50 ± 1.3
70	66.6 ± 7.9	72.7 ± 3.8	62.3 ± 3.2	62.5 ± 2.1	47.9 ± 1.5	51.3 ± 2.4	49 ± 2.6	49.7 ± 1.4
91	68.3 ± 8.5	74.7 ± 3.6	63.2 ± 2.6	65.5 ± 2	48.7 ± 1.3	50.8 ± 2.1	49.7 ± 2.8	50 ± 1.5

Water consumption (mL)								
0	100 ± 4	90 ± 2	100 ± 2	110 ± 3	100 ± 2	80 ± 2	110 ± 4	90 ± 2
14	90 ± 3	70 ± 5	110 ± 3	100 ± 1	80 ± 4	80 ± 1	70 ± 5	90 ± 2
42	90 ± 2	110 ± 3	90 ± 1	100 ± 2	100 ± 3	80 ± 4	90 ± 1	90 ± 3
70	110 ± 3	110 ± 1	100 ± 5	100 ± 3	100 ± 2	100 ± 1	90 ± 2	110 ± 3
91	100 ± 4	100 ± 2	110 ± 1	100 ± 4	100 ± 1	110 ± 2	100 ± 2	100 ± 3

*Data presented as mean ± standard deviation (*n* = 5). Significantly different from control: *P* < 0.05.

^a^Control group received 1 mL distilled water.

^b^Sample group received three doses of green mussel formulation (2.0, 1.0, and 0.5 g/kg rat).

**Table 2:** Hematology analyses data of male and female rats administered the green mussel formulation after subchronic (90 days) toxicity studies.

Treatments		Lymphocytes (mm^3^)	Eosinophils (mm^3^)	Neutrophils (mm^3^)	HGB (g/dL)	WBC (mm^3^)	RBC (10^6^/cmm)	Platelet (10^5^/cmm)
Male	Control^a^	5828.60 ± 159.14	998.20 ± 62.90	3213.20 ± 17.13	15.44 ± 0.05	10040 ± 58.6	7.87 ± 0.07	5.78 ± 0.06
	2.0 g/kg^b^	4638.00 ± 154.50	752.60 ± 31.57	2329.40 ± 83.04*	14.60 ± 0.64	7720 ± 26.70	7.41 ± 0.29	4.64 ± 0.38
	1.0 g/kg^b^	3643.80 ± 187.47*	618.60 ± 33.24*	2017.60 ± 88.02*	14.34 ± 0.73	6280 ± 30.90	7.29 ± 0.58	5.08 ± 0.04
	0.5 g/kg^b^	4912.20 ± 114.07	773.60 ± 15.38	2754.20 ± 78.98	14.64 ± 0.70	8440 ± 174.70	7.27 ± 0.08	5.78 ± 0.06

Female	Control^a^	5220.40 ± 231.0	888.80 ± 37.78	2730.80 ± 80.31	13.38 ± 0.94	8840 ± 148.9	6.37 ± 0.47	6.06 ± 0.03
	2.0 g/kg^b^	3856.40 ± 167.93*	645.40 ± 28.94	2298.20 ± 114.25	14.12 ± 0.44	6800 ± 97.7	6.97 ± 0.03	5.78 ± 0.08
	1.0 g/kg^b^	4975.20 ± 269.33	770.00 ± 31.75	1974.80 ± 83.87*	13.86 ± 0.83	7720 ± 107.5	6.86 ± 0.21	5.30 ± 0.07
	0.5 g/kg^b^	2122.80 ± 101.52*	321.20 ± 14.67*	1096.00 ± 50.93*	13.54 ± 0.53	3540 ± 101.7*	6.77 ± 0.07	5.64 ± 0.06

*Data presented as mean ± standard deviation (*n* = 5). Significantly different from control: *P* < 0.05.

^a^Control group received 1 mL distilled water.

^b^Sample group received three doses of green mussel formulation (2.0, 1.0, and 0.5 g/kg rat).

HGB: hemoglobin; WBC: total white blood cell count; RBC: red blood cell.

**Table 3:** Serum biochemical analysis data of male and female rats administered the green mussel formulation after subchronic (90 days) toxicity studies.

		SGOT	SGPT	ALP	Bilirubin	Total protein	Albumin	Globulin	A/G ratio	Urea	Creatinine
		(U/L)	(U/L)	(U/L)	(mg/dL)	(g/dL)	(g/dL)	(g/dL)		(mg/dL)	(mg/dL)
Male	Control^a^	182.00 ± 3.14	68.40 ± 1.5	356.20 ± 2.84	0.20 ± 0.01	7.82 ± 0.22	3.50 ± 0.07	4.32 ± 0.42	0.81	46.80 ± 0.96	0.68 ± 0.08
	2.0 g/kg^b^	188.60 ± 1.5	73.80 ± 10.53	254.40 ± 3.18*	0.20 ± 0.07	7.44 ± 0.16	3.54 ± 0.03	3.90 ± 0.02	0.91	50.80 ± 0.15	0.62 ± 0.04
	1.0 g/kg^b^	171.00 ± 14.93	78.40 ± 0.02	332.80 ± 2.49	0.18 ± 0.04	7.06 ± 0.06	3.54 ± 0.26	3.52 ± 0.34	1.02	42.00 ± 0.54	0.62 ± 0.08
	0.50 g/kg^b^	146.00 ± 20.73*	68.80 ± 0.42	254.40 ± 2.44*	0.18 ± 0.04	7.44 ± 0.26	3.54 ± 0.41	3.90 ± 0.51	0.93	32.00 ± 0.87*	0.62 ± 0.13
Female	Control^a^	195.40 ± 10.01	86.40 ± 1.57	267.40 ± 23	0.22 ± 0.04	7.52 ± 0.26	4.14 ± 0.21	3.38 ± 0.08	1.23	77.00 ± 5.94	0.78 ± 0.03
	2.0 g/kg^b^	132.60 ± 3.04*	63.40 ± 1.99	340.20 ± 4.94	0.16 ± 0.05	7.22 ± 0.21	3.94 ± 0.27	3.28 ± 0.3	1.20	49.00 ± 2.16*	0.56 ± 0.09
	1.0 g/kg^b^	161.25 ± 30.13	64.00 ± 0.81	344.75 ± 5.01	0.20 ± 0.01	7.72 ± 0.02	4.08 ± 0.2	3.64 ± 0.18	1.12	55.80 ± 0.95	0.68 ± 0.04
	0.50 g/kg^b^	145.20 ± 10.96*	65.60 ± 1.35	287.60 ± 3.02	0.20 ± 0.02	7.16 ± 0.05	4.22 ± 0.03	2.94 ± 0.06	1.45	56.20 ± 0.03	0.68 ± 0.11

*Data presented as mean ± standard deviation (*n* = 5). Significantly different from control: *P* < 0.05.

^a^Control group received 1 mL distilled water.

^b^Sample group received three doses of green mussel formulation (2.0, 1.0, and 0.5 g/kg rat).

SGOT: serum glutamic oxaloacetic transaminase; SGPT: serum glutamic pyruvic transaminase; ALP: alkaline phosphatase; A/G ratio: albumin/globulin ratio.

**Table 4:** Serum biochemical analysis data of male and female rats administered the green mussel formulation after subchronic (90 days) toxicity studies.

		Na^+^	K^+^	Cl^+^	HCO_3_^+^	Cholesterol	Triglycerides	HDL	LDL	VLDL
		(m·mol/L)	(m·mol/L)	(m·mol/L)	(m·mol/L)	(mg/dL)	(mg/dL)	(mg/dL)	(mg/dL)	(mg/dL)
Male	Control^a^	147.80 ± 0.66	5.50 ± 0.35	105.22 ± 0.47	25.80 ± 0.84	77.40 ± 0.19	137.50 ± 1.01	32.80 ± 0.42	18.60 ± 0.36	26.00 ± 0.89
	2.0 g/kg^b^	145.72 ± 2.96	6.05 ± 0.96	104.88 ± 1.19	27.20 ± 1.09	74.20 ± 0.72	138.80 ± 1.8	34.60 ± 0.14	11.80 ± 0.21	27.80 ± 0.95
	1.0 g/kg^b^	144.84 ± 0.34	6.24 ± 0.83	104.48 ± 0.80	26.40 ± 0.14	76.20 ± 0.73	152.80 ± 31.24	33.20 ± 0.30	13.75 ± 0.30	30.60 ± 0.06
	0.50 g/kg^b^	146.06 ± 2.22	5.15 ± 0.22	104.48 ± 0.44	27.60 ± 0.89	75.20 ± 3.70	126.00 ± 0.22	34.80 ± 0.84	15.20 ± 2.68	25.20 ± 0.79

Female	Control^a^	139.98 ± 0.68	4.88 ± 0.10	101.32 ± 1.78	25.60 ± 1.14	75.60 ± 0.44	114.20 ± 01.94	32.20 ± 0.30	20.60 ± 6.87	22.80 ± 0.30
	2.0 g/kg^b^	136.94 ± 0.73	5.03 ± 0.57	104.38 ± 0.79	27.40 ± 0.52	63.40 ± 3.36	123.40 ± 0.21	31.00 ± 0.71	7.80 ± 0.49	24.60 ± 0.89
	1.0 g/kg^b^	138.18 ± 0.66	5.58 ± 0.61	103.54 ± 0.95	26.60 ± 0.89	75.20 ± 0.68	140.60 ± 0.35	32.80 ± 0.08	16.40 ± 0.28	26.00 ± 0.64
	0.50 g/kg^b^	141.14 ± 0.59	5.85 ± 0.88	104.84 ± 0.83	27.20 ± 0.84	72.80 ± 0.03	145.60 ± 0.70	29.20 ± 1.64	14.40 ± 0.91	29.20 ± 0.15

Data presented as mean ± standard deviation (*n* = 5).

^a^Control group received 1 mL distilled water.

^b^Sample group received three doses of green mussel formulation (2.0, 1.0, and 0.5 g/kg rat).

HDL: high-density lipoprotein; LDL: low-density lipoprotein; VLDL: very low-density lipoprotein.
